# Risk of Cardiovascular Disease and Cancer in Patients Initiating JAK Versus IL‐4/‐13 Inhibitors for Atopic Dermatitis

**DOI:** 10.1111/ijd.17815

**Published:** 2025-04-28

**Authors:** Sizheng Steven Zhao, Gema Hernandez, Uazman Alam

**Affiliations:** ^1^ Centre for Musculoskeletal Research The University of Manchester Manchester UK; ^2^ NIHR Manchester Biomedical Research Centre Manchester University NHS Foundation Trust Manchester UK; ^3^ TriNetX LLC Cambridge Massachusetts USA; ^4^ Institute of Life Course and Medical Sciences University of Liverpool Liverpool UK; ^5^ Department of Medicine, University Hospital Aintree Liverpool University NHS Foundation Trust Liverpool UK

**Keywords:** cardiovascular disease, dupilumab, eczema, malignancy, tofacitinib, upadacitinib

1

JAK inhibitors (JAKi) are highly effective for treating atopic dermatitis (AD). However, concerns about cardiovascular and cancer risk persist, as their elevated risk was observed in the ORAL surveillance trial of rheumatoid arthritis (RA) [1]. While the RA and AD populations likely differ, AD is independently associated with an increased risk of cardiovascular disease and cancer compared with the general population [2, 3]. We aimed to compare the risk of cardiovascular disease and cancer between individuals starting systemic JAKi versus IL‐4/‐13 inhibitors (IL‐4/‐13i).

We conducted a cohort study utilizing electronic health records from healthcare organizations predominantly in North America. We included adults (aged over 18 years) with an ICD‐10 code for atopic dermatitis who initiated treatment with either a JAKi (tofacitinib, upadacitinib, and abrocitinib) or an IL‐4/‐13i (dupilumab, lebrikizumab, and tralokinumab). The primary outcomes were: (1) coronary artery disease or stroke and (2) any cancer. Secondary outcomes included the following: (1) coronary artery disease, (2) stroke, (3) skin cancer, and (4) herpes zoster or conjunctivitis as control outcomes. Code lists are available in the (doi:10.17632/2zfp2swz8f.1).

The time from drug initiation to each outcome was compared between the two exposure groups using 1:1 propensity score (PS)‐matched Cox proportional hazards models. PS matching included age at the index date, sex, ethnicity (White vs. others), and the following covariates assessed within 12 months prior to the index date: body mass index (BMI), C‐reactive protein level (CRP), ischemic heart disease, cerebrovascular disease, cardiovascular risk factors, cancers, and proxies of AD severity. Individuals were censored at the date of their last health record entry or the end of follow‐up, whichever came first. Those who initiated any drug in the other exposure group during the follow‐up period were excluded. We conducted analyses with 1‐, 3‐, or 5‐year follow‐up to assess potential bias from differential follow‐up times across exposure groups. We performed sensitivity analyses: (1) excluding individuals with a prior history of each outcome or (2) excluding events in the first month of follow‐up to reduce reverse causation.

A total of 1978 JAKi were matched to 1978 IL‐4/‐13i initiators. The mean follow‐up time was generally longer in the IL‐4/‐13i group (e.g., 291 vs. 264 days for the 1‐year analysis). The numbers of each outcome event are shown in Figure 1. All potential confounders were adequately balanced (Table 1). There was no statistical evidence to support a difference in cardiovascular disease (HR 1.41; 95% CI 0.78, 2.56), any cancers (HR 0.81; 95% CI 0.60, 1.08), or skin cancers (1.07; 95% CI 0.61, 1.89) between the two drug groups. The results were similar over 1 to 5 years of follow‐up and in sensitivity analyses. Additional data are provided in the (doi:10.17632/2zfp2swz8f.1). JAKi was associated with a higher risk of zoster and a lower risk of conjunctivitis (Figure 1).

**FIGURE 1:**
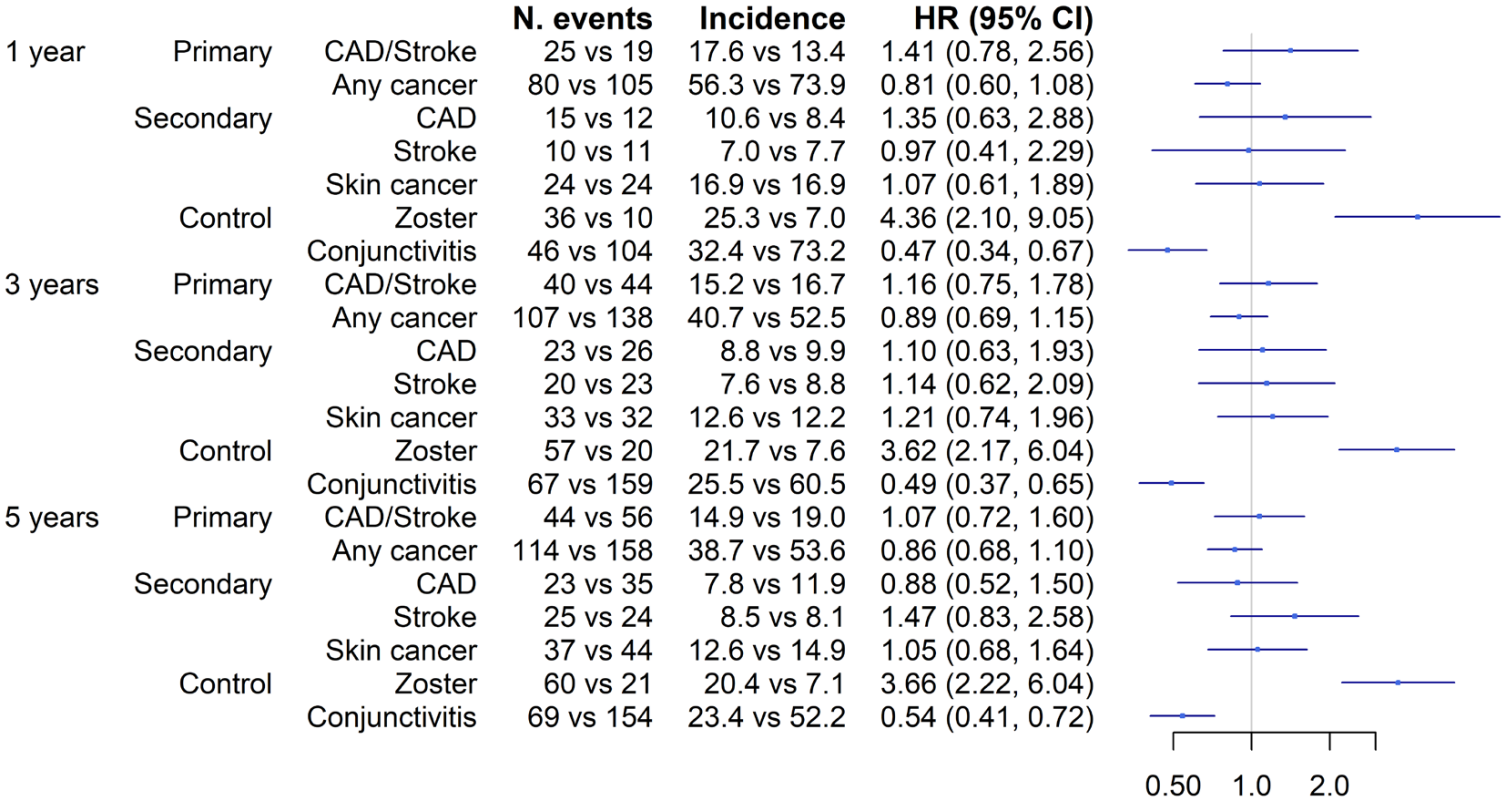
Cardiovascular and cancer risk in adults with atopic dermatitis initiating JAK versus IL‐4/‐13 inhibitors at 1, 3, and 5 years of follow‐up. Results are shown as hazard ratios and 95% confidence intervals. Incidence shown as per 1000 person years. CAD, coronary artery disease.

**TABLE 1:** Baseline characteristics before and after propensity score matching.

	Before matching			After matching		
	JAKi	IL‐4/‐13i	SMD	JAKi	IL‐4/‐13i	SMD
*N*	2001	17,216		1978	1978	
Age (years)	45.8 (18.3)	46.7 (19.2)	0.047	45.8 (18.3)	45.5 (18.6)	0.014
Female	1225 (61.2%)	9648 (56.0%)	0.105	1206 (61.0%)	1207 (61.0%)	0.001
White	1084 (54.2%)	9144 (53.1%)	0.021	1071 (54.1%)	1093 (55.3%)	0.022
CRP (mg/L)	17.9 (38.5)	8.6 (19.7)	0.305	14.5 (43.8)	13.4 (23.4)	0.032
BMI (kg/m^2^)	29.5 (7.1)	29.3 (7.4)	0.026	29.3 (6.8)	30.3 (8.2)	0.138
Hypertension	64 (3.2%)	524 (3.0%)	0.009	61 (3.1%)	49 (2.5%)	0.037
Ischemic heart diseases	10 (0.5%)	86 (0.5%)	< 0.001	10 (0.5%)	10 (0.5%)	< 0.001
Cerebrovascular diseases	10 (0.5%)	46 (0.3%)	0.038	10 (0.5%)	10 (0.5%)	< 0.001
COPD	14 (0.7%)	99 (0.6%)	0.016	14 (0.7%)	10 (0.5%)	0.026
Tobacco use	10 (0.5%)	21 (0.1%)	0.068	10 (0.5%)	10 (0.5%)	< 0.001
Overweight/obesity	29 (1.4%)	208 (1.2%)	0.021	29 (1.5%)	21 (1.1%)	0.036
Type 2 diabetes mellitus	39 (1.9%)	246 (1.4%)	0.04	38 (1.9%)	27 (1.4%)	0.044
Dislipidemia	67 (3.3%)	395 (2.3%)	0.064	67 (3.4%)	50 (2.5%)	0.051
Cancers	45 (2.2%)	676 (3.9%)	0.097	45 (2.3%)	41 (2.1%)	0.014
Corticosteroids, weak	87 (4.3%)	821 (4.8%)	0.02	82 (4.1%)	62 (3.1%)	0.054
Corticosteroids, moderately potent	225 (11.2%)	3327 (19.3%)	0.226	225 (11.4%)	222 (11.2%)	0.005
Corticosteroids, potent	200 (10.0%)	2458 (14.3%)	0.131	197 (10.0%)	170 (8.6%)	0.047
Corticosteroids, very potent	100 (5.0%)	1461 (8.5%)	0.139	99 (5.0%)	92 (4.7%)	0.017
Corticosteroids, others	284 (14.2%)	3897 (22.6%)	0.219	282 (14.3%)	268 (13.5%)	0.02
Calcineurin inhibitors	88 (4.4%)	1758 (10.2%)	0.225	87 (4.4%)	89 (4.5%)	0.005
Methotrexate	61 (3.0%)	211 (1.2%)	0.126	54 (2.7%)	52 (2.6%)	0.006
Azathioprine	10 (0.5%)	18 (0.1%)	0.072	10 (0.5%)	10 (0.5%)	< 0.001
Mycophenolic acid	10 (0.5%)	10 (0.1%)	0.084	10 (0.5%)	10 (0.5%)	< 0.001
Antibacterials	135 (6.7%)	1090 (6.3%)	0.017	130 (6.6%)	114 (5.8%)	0.034
Dupilumab	0	17,006 (98.8%)	na	0	1949 (98.5%)	na
Tralokinumab	0	210 (1.2%)	na	0	29 (1.5%)	na
Abrocitinib	160 (8.0%)	0	na	160 (8.1%)	0	na
Upadacitinib	1366 (68.3%)	0	na	1358 (68.7%)	0	na
Tofacitinib	481 (24.0%)	0	na	466 (23.6%)	0	na

*Note:* Data shown as *n* (%) or mean (standard deviation). The number of individual exposure drugs combined may exceed the total when individuals start a second drug after only brief exposure to the first.

Abbreviations: BMI, body mass index; COPD, chronic obstructive pulmonary disease; CRP, C‐reactive protein; IHD, ischemic heart disease; SMD, standardized mean difference.

In this large real‐world study evaluating JAKi safety among adults with AD, we found no evidence of increased cardiovascular or cancer risk compared to IL‐4/‐13i. These findings are reassuring and align with AD trials [4] and a substantial body of observational evidence from RA [5]. Although this is the largest analysis to date, the relatively small number of observed events limited our statistical power, highlighting the need for replication and ongoing pharmacovigilance. The main limitation is the inability to accurately ascertain the drug cessation date using administrative data and the differences in follow‐up time between exposure groups. However, results were similar across different durations of follow‐up. As with all observational studies, residual confounding may bias our results. Nonetheless, reassurance comes from using IL‐4/‐13i as an active comparator and from the use of control outcomes. In conclusion, JAK inhibitors were not associated with higher cardiovascular or cancer risk in adults with AD when compared to IL‐4/‐13i.

## Conflicts of Interest

S.S.Z. has received support from Alfasigma, Eli Lilly, Novartis, and UCB for conference attendance and honoraria from AbbVie, Novartis, and UCB for speaking at non‐promotional educational events, none of which are related to the current research. G.H. is an employee of TriNetX. U.A. has received honoraria from Eli Lilly, Procter & Gamble, Viatris, Grunenthal, and Sanofi for educational meetings and funding for attendance at educational meetings from Daiichi Sankyo and Sanofi, none of which are related to the current research. U.A. has also received investigator‐led funding from Procter & Gamble and is a council member of the Royal Society of Medicine's Vascular, Lipid & Metabolic Medicine Section, neither of which is related to the current research.
